# Two Forms of Activation-Induced Cytidine Deaminase Differing in Their Ability to Bind Agarose

**DOI:** 10.1371/journal.pone.0008883

**Published:** 2010-01-27

**Authors:** Mirjam Metzner, Wolfgang Schuh, Edith Roth, Hans-Martin Jäck, Matthias Wabl

**Affiliations:** 1 Nikolaus-Fiebiger Center, Department of Internal Medicine III, Division of Molecular Immunology, University of Erlangen-Nürnberg, Erlangen, Germany; 2 Department of Microbiology and Immunology, University of California San Francisco, San Francisco, California, United States of America; University of Minnesota, United States of America

## Abstract

**Background:**

Activation-induced cytidine deaminase (AID) is a B-cell-specific DNA mutator that plays a key role in the formation of the secondary antibody repertoire in germinal center B cells. In the search for binding partners, protein coimmunoprecipitation assays are often performed, generally with agarose beads.

**Methodology/Principal Findings:**

We found that, regardless of whether cell lysates containing exogenous or endogenous AID were examined, one of two mouse AID forms bound to agarose alone.

**Conclusions/Significance:**

These binding characteristics may be due to the known post-translational modifications of AID; they may also need to be considered in coimmunoprecipitation experiments to avoid false-positive results.

## Introduction

Activation-induced cytidine deaminase (AID) is a B-cell-specific DNA mutator that plays a key role in the formation of the secondary antibody repertoire in germinal center B cells. AID is the master protein for somatic hypermutation and class switching of immunoglobulin genes, both of which are initiated by the relatively small 24-kDa AID enzyme [1], [2]. AID is almost exclusively expressed in activated germinal center B cells [2]. With its nuclear localization signal and a nuclear export signal at the N-terminus and C-terminus, respectively, AID shuttles between the cytoplasm and the nucleus [3], [4].

It is still a mystery how AID mediates two processes as distinct as hypermutation and class switching, but it is thought that specific cofactors interact with AID for control. While the N-terminal domain of AID is required for hypermutation, the C-terminal domain is essential for class switching but not for hypermutation [5]–[8]. Factors interacting with AID might also be needed to restrict the mutator activity of AID to the immunoglobulin loci and protect genome integrity: For example, constitutively expressed AID leads to T cell lymphomas and lung micro-adenomas in transgenic mice [9], and AID expression has been linked to oncogenic translocations [10], [11] and inflammation-associated cancer [12]–[14]. But the known AID-interacting proteins—replication protein A [15], protein kinase A [16], [17], DNA-dependent protein kinase catalytic subunit [18], and the spliceosome-associated factor CTNNBL1 [19]—do not have the specificity needed for these functions.

In a widely used method to find factors binding to a protein of interest, specific antibodies in combination with agarose beads are added to cell lysates to coimmunoprecipitate these factors. Commonly, the agarose beads are coupled with protein A or G, both of which are bacterial cell wall components that bind to the constant region of the heavy chain of antibodies. But it is well known that some proteins tend to stick to the protein A or G, or to the agarose itself. This problem can be avoided by performing a pre-clearing step before the actual coimmunoprecipitation. However, sometimes the protein of interest itself binds to the beads. Then coimmunoprecipitation cannot be used without modifying the protocol; or, worse, precipitated molecules give false-positive results. If binding is of low affinity, the amount of protein that adheres to the beads in a pre-clearing step will be small, and there will be enough protein left to be precipitated in a subsequent step, using specific antibody to either the protein of interest or the putative binding partner. Alternatively, there are two forms of the protein of interest—one binding to agarose (with high affinity), the other not. We found that this is the case for AID, i.e., that there are two forms that bind agarose differently.

## Materials and Methods

### Ethics Statement

All experiments with mice were performed following the protocol AN077866-03B, most recently re-approved by the IACUC on March 2009, as a yearly renewal. UCSF's animal house abides by all governmental regulations and standards, Animal Welfare Assurance Number A3400-01.

### AID Induction in Primary B Cells

Primary splenic cells from BALB/c mice were cultured in standard medium and stimulated with 50 µg/ml LPS (Sigma) and 100 ng/ml IL-4 (R&D Systems). After 4 days the activated B cells were harvested and subjected to agarose binding assays. As controls, B cells from age-and sex-matched AID^−/−^ BALB/c mice were cultured under the same conditions.

### AID Induction in Stable HeLa Cell Transfectants

HeLa cells, stably transfected with a doxycycline-inducible mouse wild-type AID construct [20] were induced with 1 µg/ml doxycycline. After 24 hours, AID expressing cells were harvested for further experiments.

### AID Transfection of Phoenix Cells

Ecotropic-package Phoenix cells (of human embryonic kidney cell origin) were transiently transfected (Lipofectamine; Invitrogen) with the retroviral pCru plasmid [21] encoding the mouse wild-type AID protein with or without N-terminal Flag-tag. As a control, cells were transfected with the pCru plasmid encoding GFP with or without N-terminal Flag-tag. Seventy-two hours after transfection the cells were harvested and subjected to agarose binding assays.

### Agarose Binding Assay

LPS-and IL-4-stimulated wild-type and AID^−/−^ B cells and AID-and GFP-transfected Phoenix cells were harvested and washed twice with ice-cold PBS. Cells were resuspended in ice-cold Triton lysis buffer (0.5% Triton X-100, 150 mM NaCl, 5 mM EDTA, 50 mM Tris pH 7.4, 15 mM NaN_3_, 1 mM PMSF, protease inhibitor cocktail set V (Calbiochem)), incubated on ice for 30 minutes, and centrifuged at 11,000 g for 15 minutes at 4°C. The supernatants were collected as the cleared lysates and used for the agarose binding assay: Phoenix-AID or Phoenix-GFP lysate (5×10^5^ cells in 100 µl of lysis buffer per sample) or LPS-and IL-4-stimulated wild-type and AID^−/−^ B cells (1×10^7^ in 100 µl of lysis buffer per sample) were incubated with 30 µl of agarose beads (1st round) for 1 hour at 4°C on an orbital shaker. The unbound lysate was incubated a second time with 30 µl of agarose beads (2nd round) for 1 hour at 4°C on an orbital shaker. The unbound lysates were saved, and 1st and 2nd round beads were washed five times with 500 µl of ice-cold PBS and centrifuged after each washing step at 5,000 g for 3 minutes at 4°C. For the agarose binding assay in the presence of RNase (only in the case of transfected, exogenous AID), the samples were treated with RNase A (Roche) at concentrations of 0, 1 µg/ml (low), 100 µg/ml (medium), or 1 mg/ml (high) RNase for 1 hour at room temperature before incubation with agarose beads. The washed 1st and 2nd round beads and the unbound lysate (supernatant) were mixed with SDS loading buffer and heated for 10 minutes at 94°C before SDS-PAGE analysis and subsequent transfer to a nitrocellulose membrane. The membrane was incubated with primary and secondary antibodies, followed by chemiluminescence.

For the detection of mouse AID, the mouse monoclonal anti-mouse AID antibody AIDA 94.16 was used together with a corresponding HRP-coupled secondary antibody. Antibody AIDA 94.16 was generated by immunizing BALB/c mice with a mix of three different mouse AID peptides: peptide 1, AID aa position 13–27, YHFKNVRWAKGRHET; peptide 3a, AID aa position 61–74, FLRYISDWDLDPGR; and peptide 4, AID aa position 116–131, CEDRKAEPEGLRRLHR. Hybridoma supernatants were tested on ELISA plates coated with mouse AID peptides and by western blot analyses using cell lysates with endogenous or exogenous (transfected) mouse AID. Positive candidate clones were subcloned via limiting dilution and tested again for AID specificity.

The agarose binding assay using high molecular mass (HMM) and low molecular mass (LMM) AID was performed as described above; instead of unfractionated cell lysates, 100 µl of the relevant 1 ml size exclusion chromatography fractions were used. The agarose binding assay using mouse wild-type AID expressed in E. coli was performed as described above; instead of cell lysates, 2 µg purified AID with N-terminal MBP-tag and, as a control, 2 µg purified MBP were used. For the detection of AID-MBP and MBP, the mouse monoclonal anti-MBP antibody M1321 (Sigma) was used together with a corresponding HRP-coupled secondary antibody.

As a positive control for the RNase treatment, 1 µg RNA was incubated for 30 minutes at room temperature in the absence (\) and presence of RNase A at various concentrations: low (L) (1 µg/ml), medium (M) (100 µg/ml), or high (H) (1 mg/ml). The degradation of the RNA was analyzed on a 1% agarose gel.

The following beads were used for agarose binding assays: For wild-type and AID^−/−^ B cell lysates: protein G agarose (Thermo Scientific, cat. #20398). For AID-and GFP-transfected Phoenix cell lysates: protein G agarose (Thermo Scientific, cat. #20398), protein A agarose (Thermo Scientific, cat. #20333), protein G Sepharose (Sigma, cat. #P3296), calmodulin Sepharose (GE Healthcare, cat. #17-0529-01), IgG Sepharose (GE Healthcare, cat. #17-0969-01); anti-IgM agarose (Sigma, cat. #A4540), anti-Flag agarose (Sigma, cat. #A2220), and anti-IgY agarose (Aves Labs, cat. #P-1010). (Sepharose is a trade name of agarose with some changes in the charge of the polysaccharides.)

### Immunoprecipitation

Immunoprecipitation experiments were performed following the agarose binding assay protocol with addition of an antibody-antigen precipitation step. Between 1st and 2nd round beads (protein G agarose, Invitrogen, cat. #15920-010) the lysate was incubated with 5 µg precipitating antibody overnight at 4°C (mouse monoclonal anti-AID antibody AIDA 94.16 or mouse monoclonal anti-Flag antibody (Sigma, cat. #F1804)). For western blotting of AID, the biotinylated anti-AID antibody AIDA 94.16 was used together with a Streptavidin HRP-coupled secondary antibody; for western blotting of Flag-tagged proteins, a mouse monoclonal anti-Flag antibody (Sigma, cat #F1804).

## Results and Discussion

### Two AID Variants within a Cell: One Binds to Agarose, the Other One Does Not

In our studies to precipitate AID from cell lysates with antibody to AID, we observed that mouse AID bound to agarose beads that we added to the lysate in a pre-clearing step. Because such binding stymied our attempts to immunoprecipitate AID, we investigated this binding in greater detail. We considered two possibilities, and combinations thereof: (i) AID binds to agarose (directly or indirectly) with low affinity, in which case not all AID will be removed from the cell lysate in the pre-clearing step. It will therefore still be present in the precipitate generated in the second round of binding, when agarose is added again; (ii) There are two forms of AID, one that binds to agarose with high affinity and one that does not.

**Figure 1:**
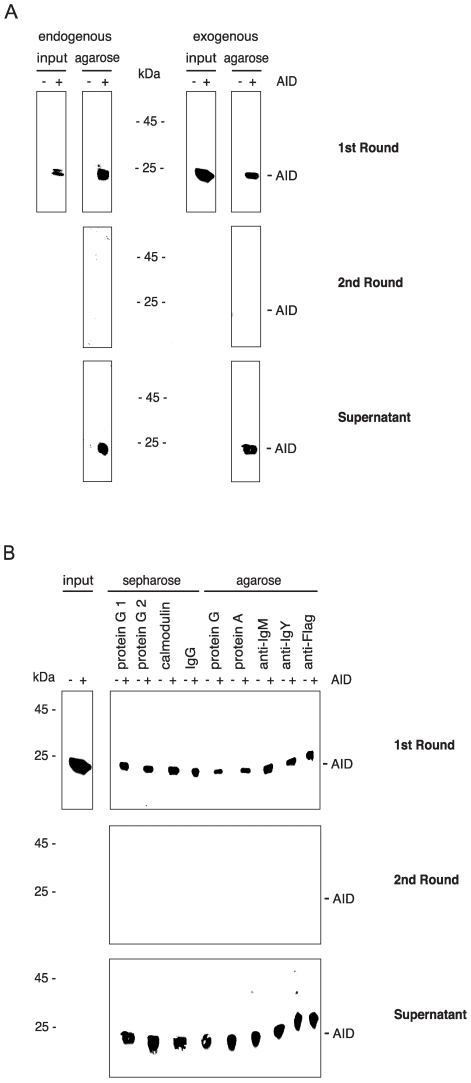
Two AID variants with different agarose binding. Cell lysates were incubated with agarose beads (1st Round). The unbound lysate was incubated a second time with agarose beads (2nd Round). The washed 1st and 2nd round beads and the unbound lysate after the second incubation (Supernatant) were western blotted and developed with a monoclonal anti-AID antibody. The positions of the molecular mass standards are indicated next to the blots. (A) Endogenous AID:–AID, lysate from AID-deficient cells; + AID, lysate from wild-type cells; input equivalent to 2×10^6^ cells; 1st Round, 2nd Round, and Supernatant, equivalent to 1×10^7^ cells. Exogenous AID:–AID, lysate from GFP-transfected cells; + AID, lysate from AID-transfected cells; input, 1st Round, 2nd Round, and Supernatant, all equivalent to 5×10^5^ cells. (B) Incubation with various Sepharose/agarose beads; exogenous AID only. Sepharose is a trade name of agarose with some changes in the charge of the polysaccharides. Protein G1 Sepharose and protein G2 Sepharose are protein G beads from different batches of the same catalog number.

We thus systematically performed agarose binding assays with cell lysates containing endogenous or exogenous mouse AID. The exogenous AID was produced by transfected Phoenix cells, and the endogenous AID came from mitogen-and IL-4-stimulated spleen cells from BALB/c mice. After incubating the lysates with agarose beads, we incubated the unbound lysate a second time with the beads. To test for the presence of AID, we then analyzed the total cell lysate, the cell fraction bound to agarose, and the unbound lysate (the supernatant after removal of the agarose beads) on western blots (Figure 1). Both exogenous and endogenous mouse AID strongly bound to agarose in the 1st round but not in the 2nd (Figure 1A), even though there was still much AID present (Figure 1A, Supernatant). These results imply that there are two variants of AID present within a cell, one able to bind to agarose, the other not.

**Figure 2:**
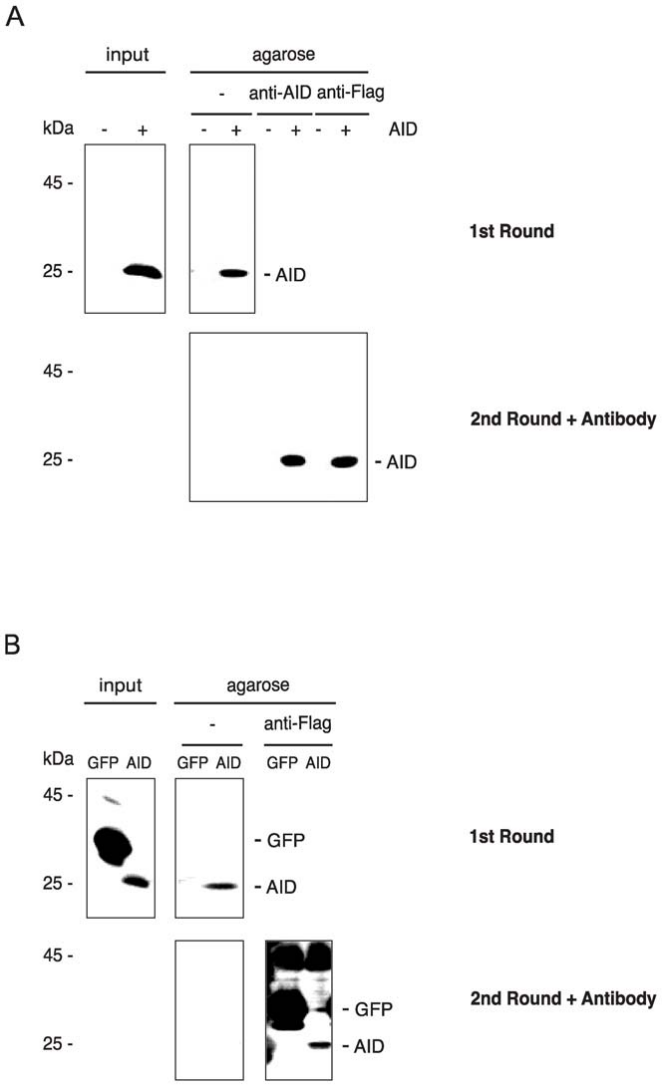
Agarose binding and immunoprecipitation of AID and GFP. Lysates of 5×10^5^ Flag-AID-and Flag-GFP-transfected cells were incubated with agarose beads (1st Round). In a second round, the unbound lysate was incubated with 5 µg anti-AID or anti-Flag antibody as indicated, and agarose beads were added (2nd Round + Antibody). The washed 1st and 2nd round beads were western blotted. The positions of the molecular mass standards are indicated next to the blots. (A) Blot developed with a monoclonal anti-AID antibody. (B) Blot developed with a monoclonal anti-Flag antibody.

To determine which component of the agarose beads was responsible for the binding to AID, we performed binding assays using agarose coupled with protein G, protein A, calmodulin or (irrelevant) antibody. Since AID bound to all beads (Figure 1B), it is thus the agarose to which AID binds. Noticeably, the ratio of bound to unbound AID was variable to some extent (see, for example, Figure 1A vs. Figure 1B); these differences in binding efficiency of AID to beads may be explained by technical variation between experiments, and by different binding capacity of the various beads. To confirm that part of the AID is still present in the unbound fraction after agarose incubation, we detected AID after precipitation with anti-AID and anti-Flag antibodies, respectively (Figure 2, 2nd Round + Antibody). To exclude that under the assay conditions of our experiments, any protein binds to agarose in a similar manner as AID, we performed an agarose binding assay using Flag-GFP cell lysates (Figure 2B). Unless a specific antibody to it was added (Figure 2B, 2nd Round + Antibody), GFP was not isolated from lysate with agarose (Figure 2B, 1st Round).

### AID Binding to Agarose Is Not RNase Sensitive

**Figure 3:**
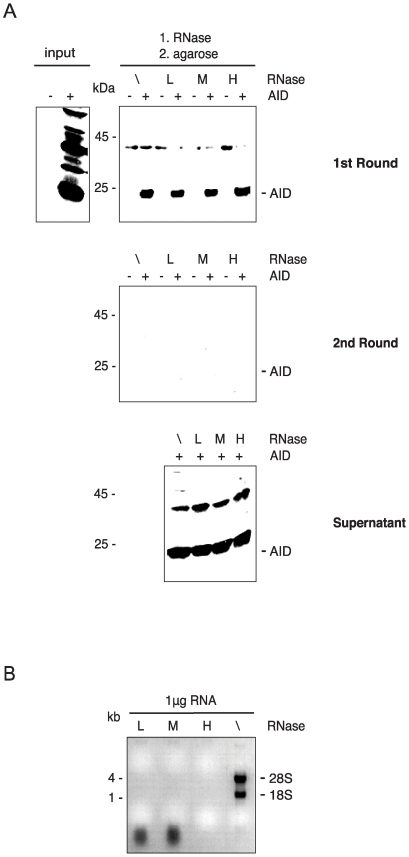
AID's binding to agarose is not RNase sensitive. (A) Agarose binding to AID in the absence (\) and presence of RNase A at various concentrations: low (L) (1 µg/ml), medium (M) (100 µg/ml), or high (H) (1 mg/ml). GFP control (–AID) or AID-transfected (+ AID) human embryonic kidney cell lysates were treated with RNase and incubated with two successive rounds of agarose beads (1st and 2nd Round). The washed 1st and 2nd round beads and the unbound lysate after the second bead incubation (Supernatant) were western blotted and probed with a monoclonal anti-AID antibody. Input, 1st Round, 2nd Round, and Supernatant, all equivalent to 5×10^5^ cells. The positions of the molecular mass standards are indicated next to the blots. (B) Incubation of 1 µg RNA in the absence (\) and presence of RNase A at various concentrations: low (L) (1 µg/ml), medium (M) (100 µg/ml), or high (H) (1 mg/ml). The positions of the size standards and the 18S and 28S rRNA are indicated next to the agarose gel.

AID produced in insect cells needs to be treated with RNase to be catalytically active [22]; therefore, AID may also interact with RNA in mammalian cells. In that case, AID may bind to agarose via the RNA. To investigate this question, we performed agarose binding assays in the presence of increasing concentrations of RNase A (Figure 3A). In these experiments, cell lysates were first incubated with RNase and then with two successive rounds of agarose (Figure 3A). To monitor RNase activity, 1 µg RNA (mainly ribosomal RNA) was incubated in the presence of increasing concentrations of RNase A (low, medium and high in Figure 3B). Because RNase treatment did not affect the binding ability of AID to agarose, we conclude that it is not the RNA that mediates it.

### Agarose Binding of High Molecular Mass (HMM) and Low Molecular Mass (LMM) AID Complexes

**Figure 4:**
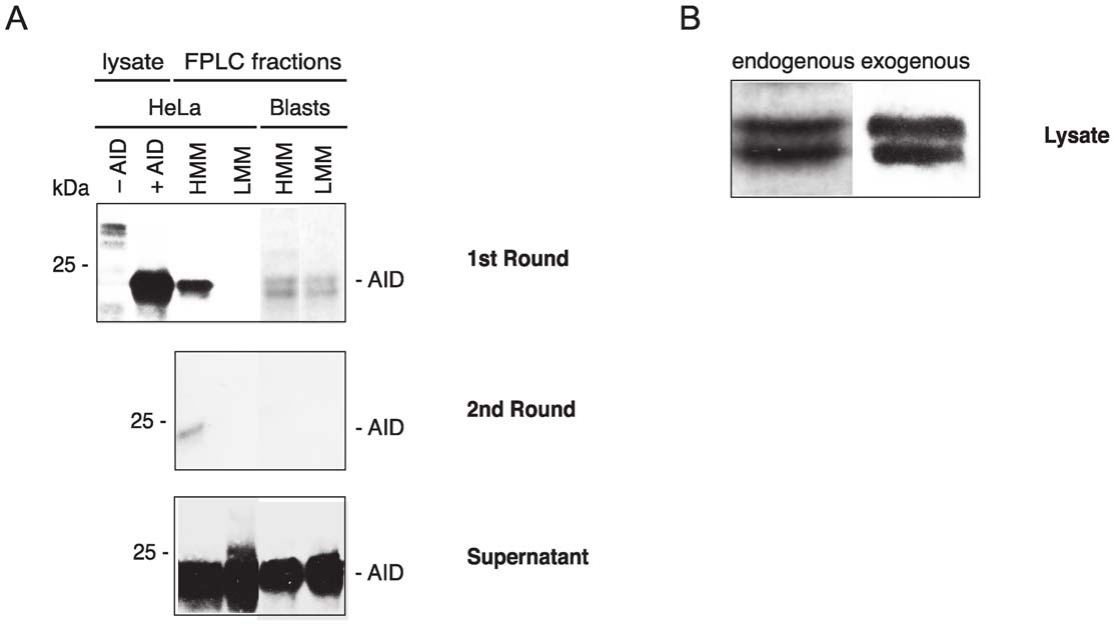
Agarose binding of high molecular mass (HMM) and low molecular mass (LMM) AID complexes. (A) AID lysate, fractionated by size exclusion chromatography, was incubated with agarose beads (1st Round). The unbound proteins were incubated a second time with agarose beads (2nd Round). The proteins recovered from the washed 1st and 2nd round beads, and the unbound proteins after the second incubation (Supernatant), were western blotted and developed with a monoclonal anti-AID antibody. The positions of the molecular mass standards are indicated next to the blots. Lysate, unfractionated lysate from HeLa (–AID) and HeLa-AID (+ AID) cells; FPLC fractions, size-fractionated HMM and LMM AID from HeLa-AID cells and primary blasts. (B) AID double band from endogenous (lysate from primary blasts), and exogenous (lysate from HeLa-AID cells) AID.

To investigate whether the different agarose binding behavior of AID could be explained by the presence or absence of additional proteins that AID forms complexes with, we performed size exclusion chromatography of AID positive cell lysates and did an agarose binding assay with the fractionated high molecular mass (HMM) and low molecular mass (LMM) AID protein complexes (Figure 4A). In the case of exogenous AID expressed in HeLa cells, the HMM AID bound to agarose, again with part of it not binding (Figure 4A, HeLa HMM); the LMM AID however did not bind at all (Figure 4, HeLa LMM). In the case of endogenous AID expressed in stimulated B cells, both the HMM and the LMM AID showed one part of AID binding, the other one not binding to agarose (Figure 4A, Blasts). Although sometimes not visible due to large amounts of protein present on the membranes and long exposure of the films, both endogenous and exogenous AID are usually represented by a double band (Figure 4B); and both proteins of the AID doublet bind equally well to agarose (Figure 4A).

**Figure 5:**
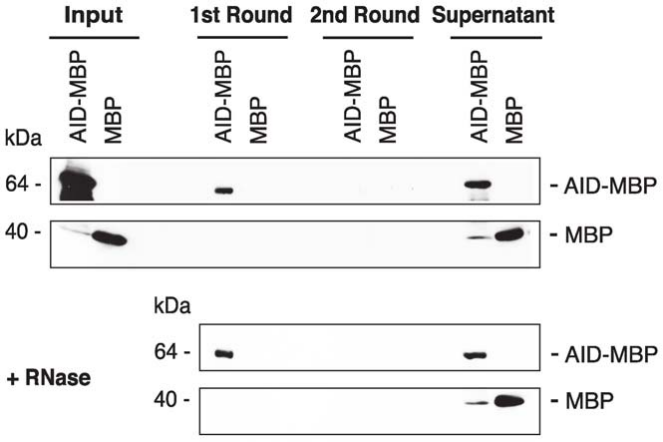
Agarose binding of AID expressed in E. coli. 2 µg of purified murine AID-MBP and MBP, respectively, (Input) were treated with 100 µg/ml RNase (lower panel) or not (upper panel) and incubated with two successive rounds of agarose beads (1st and 2nd Round). The proteins recovered from the washed 1st and 2nd round beads, and the unbound proteins after the second bead incubation (Supernatant), were western blotted and probed with a monoclonal anti-MBP antibody. The positions of the molecular mass standards are indicated next to the blots.

For the experiments described above we used mouse AID expressed in eukaryotic cells, but we also tested mouse AID expressed in E. coli. The AID with an N-terminal maltose binding protein (MBP)-tag behaved like the AID expressed in mouse cells: one form bound to agarose, another did not (Figure 5, 1st, 2nd Round, respectively). MBP alone did not bind to agarose (Figure 5, 1st and 2nd Round), confirming that it is the AID part of the AID-MBP fusion protein that binds. Once again, binding to agarose was not affected by RNase treatment of the sample beforehand (Figure 5, lower panel).

### Conclusions

Our finding that one form of AID strongly binds to agarose beads complicates the interpretation of coprecipitation experiments done to identify protein interaction partners of AID within a cell. Especially when one does not incubate the protein lysate with agarose beads alone, one cannot be sure about an actual interaction. On the upside, two AID variants can be distinguished by way of their difference in binding to agarose; endogenous and exogenous AID display the same binding characteristic toward agarose. It did not make a difference whether AID was expressed in bacteria, or mouse and human cells; or whether AID was part of the high or low molecular mass complex in activated B lymphocytes. Nor did the two proteins represented by the AID doublet differ in this regard. The two AID forms—binding and non-binding—could represent different post-translational modifications. Some of the modifications determine the type of AID activity, whether class switching or hypermutation [16], [17], [23]–[25]. But because AID expressed in bacteria did not show a difference, the modifications need to be restricted to those also found in bacteria.
